# Are subjective sleepiness and sleep quality related to prospective memory?

**DOI:** 10.1186/s41235-019-0199-7

**Published:** 2020-02-07

**Authors:** Mateja F. Böhm, Ute J. Bayen, Marie Luisa Schaper

**Affiliations:** Institute for Experimental Psychology, Heinrich-Heine-Universität Düsseldorf, Gebäude 23.02, Universitätsstraße 1, 40225 Düsseldorf, Germany

**Keywords:** Prospective memory, Body posture, Sleepiness, Sleep quality, Multinomial modeling

## Abstract

Event-based prospective memory (PM) involves carrying out intentions when specific events occur and is ubiquitous in everyday life. It consists of a prospective component (remembering *that* something must be done) and a retrospective component (remembering *what* must be done and *when*). Subjective sleep-related variables may be related to PM performance and an attention-demanding prospective component. In two studies, the relationship of subjective sleepiness and subjective sleep quality with both PM components was investigated with a laboratory PM task and separation of its components via Bayesian multinomial processing tree modeling. In Study 1, neither component of PM was related to naturally occurring subjective sleepiness or sleep quality. In Study 2, sleepiness was experimentally increased by placing some participants in a supine body posture. Testing participants in upright vs. supine posture affected neither PM component. However, body posture moderated the relationship between subjective sleep quality and the prospective component: In supine posture, subjective sleep quality tended to be more positively related to the prospective component. Overall, neither subjective sleepiness nor subjective sleep quality alone was related to PM.

## Significance

Prospective memory involves remembering to do something at the right time in the future and is frequently needed in everyday life. For instance, remembering to buy orange juice on the way home is a prospective-memory task. As prospective memory is so important in everyday life and prospective memory failures may have severe consequences, researchers focus on identifying factors that may influence prospective memory. One of these factors is sleep: Many previous studies have shown that not sleeping at all or only for a short period of time impairs prospective memory. In addition, poor sleep quality impairs prospective memory. These studies predominantly used objective sleep measures. However, subjective sleep measures have been largely neglected, although previous studies suggested that subjective sleep measures are related to memory and attention. Therefore, we examined the relationship between prospective memory and subjective sleep measures. In two studies, research participants performed a prospective-memory task on a computer. Some participants did this while sitting upright and the others while lying down because lying down induces additional sleepiness. Subjective sleepiness and sleep quality were not related to prospective memory in upright posture. The different postures also did not lead to differences in prospective memory. Overall, the results suggest that subjective sleep measures are not related to prospective memory. Future studies should investigate whether the current findings also hold for people suffering from sleep disorders and excessive daytime sleepiness.

## Are subjective sleepiness and sleep quality related to prospective memory?

Prospective memory (PM) involves remembering to perform intentions at appropriate times in the future (e.g., Einstein & McDaniel, 1990). For example, one must remember to buy orange juice on the way home from work. PM is ubiquitous in daily life and important in many jobs and professions such as health care, air traffic control, and office work (e.g., Dismukes, 2012). Because of the importance of PM in daily life, it is crucial to identify variables that influence PM. One such variable is sleep. Previous studies have shown that PM benefits when sleep takes place during the retention interval (i.e., between forming and carrying out the intention), but suffers from sleep deprivation (for a review, see Leong, Cheng, Chee, & Lo, 2019). However, as of yet, subjective sleep measures have not received much attention in PM research, although complaints about poor sleep are on the rise (e.g., Kronholm et al., 2008). Therefore, in Study 1, we investigated whether subjective sleepiness and subjective sleep quality are related to PM. In Study 2, we induced subjective sleepiness by manipulating body posture. This allowed us to experimentally investigate effects of posture-induced sleepiness on PM.

The studies reported here focus on event-based PM, which involves remembering to perform planned actions when specific events occur. For instance, an administrative assistant may have to remember to forward emails regarding specific topics to his boss when such emails arrive. Event-based PM has two components (Einstein & McDaniel, 1990). The *prospective component* involves remembering *that* an intention needs to be carried out; the *retrospective component* involves remembering *what* the intention is and *when* it should be carried out. In the previous example, the administrative assistant has to remember an additional intention while checking emails (prospective component). He must also remember the topics of the emails that need to be forwarded (retrospective component). As explained in detail below, the literature suggests that the prospective and retrospective components of PM may be differentially related to subjective sleepiness and sleep quality. It was therefore important in our studies to separately measure the prospective and retrospective components of a PM task. To achieve this, we chose a computerized PM task that allowed us to obtain valid measures of the prospective and retrospective task components via a mathematical model (that we explain in the Results section of Study 1).

We will first introduce the computerized PM task paradigm we used. We will then review the literature on possible relationships of PM with subjective sleepiness and sleep quality. Following this review, we will derive differential hypotheses for the relationships of the prospective and retrospective components of PM with subjective sleepiness and sleep quality.

**Fig. 1:**
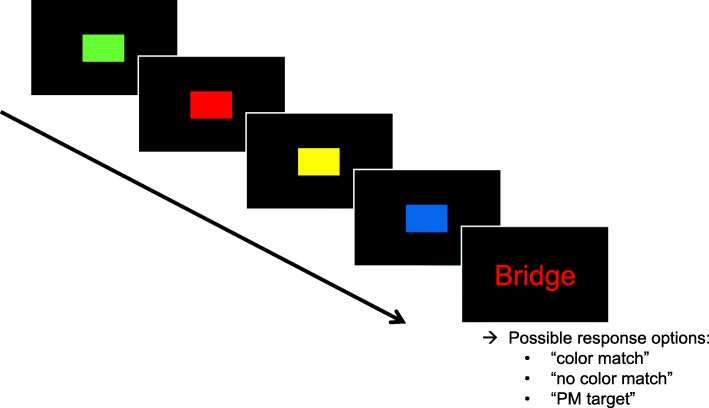
Example trial of the prospective-memory (PM) task embedded in the ongoing color-matching task. Adapted by permission from R. E. Smith and R. R. Hunt (2012). *Prospective Memory: Adult Age, Ongoing Task Difficulty, and Task Importance* [Poster presentation]. Cognitive Aging Conference, Atlanta, GA, United States

The general paradigm for computerized PM tasks involves an ongoing task with an embedded PM task (Einstein & McDaniel, 1990). Participants perform the ongoing task. Upon occurrence of a PM target event, they must interrupt the ongoing task and instead perform the PM task, then carry on with the ongoing task. Figure 1 illustrates the task paradigm used in our studies. The ongoing task was a color-matching task, in which participants indicated whether a word had the same color as one of four previously presented rectangles (cf. Smith & Bayen, 2004). The PM task was to press a specific key whenever participants encountered one of several previously studied words during the ongoing task. Critically, the prospective component of this PM task is to remember that one has to do something in addition to the ongoing color-matching task. The retrospective component of the task is to recognize PM target items and to distinguish them from distractor items.

The task is a nonfocal PM task. Nonfocal PM targets have different defining features than the ongoing task (see McDaniel & Einstein, 2007). In such tasks, participants must shift their attentional focus from features relevant for the ongoing task (e.g., word color) to features relevant for the PM task (e.g., word meaning). PM theories agree that in PM tasks that are *nonfocal* to the ongoing task, the prospective component depends on attentional processes (e.g., multiprocess framework, McDaniel & Einstein, 2000; dynamic multiprocess framework, Scullin, McDaniel, & Shelton, 2013; preparatory attentional processes and memory theory, Smith, 2003). Therefore, the prospective component of the presented task should rely on attentional processes, which is relevant for its relation with sleep variables, as explained later.

### Prospective memory and sleep

As PM plays such an important role in everyday life, much recent research has been devoted to the effects of sleep on PM. Studies have shown that sleep deprivation impairs PM (Esposito, Occhionero, & Cicogna, 2015; Grundgeiger, Bayen, & Horn, 2014; Leong, Koh, Tandi, Chee, & Lo, 2018; Occhionero, Cicogna, & Esposito, 2017), and that PM benefits when sleep takes place during the retention interval (Barner, Seibold, Born, & Diekelmann, 2016; Diekelmann, Wilhelm, Wagner, & Born, 2013a, 2013b; Leong, Koh, Chee, & Lo, 2019; Leong, van Rijn, Koh, Chee, & Lo, 2019) because presumably sleep consolidates the association between the intention and the context in which it must be carried out (Scullin & McDaniel, 2010). Furthermore, some studies showed that PM is influenced by sleep characteristics such as sleep quality (Fabbri, Tonetti, Martoni, & Natale, 2014) and sleep stage (Diekelmann et al., 2013b; Leong, Koh, et al., 2019; Scullin et al., 2019). Taken together, these studies suggest that objective measures of sleep and sleep quality affect PM. Subjective sleep measures, on the other hand, have been used less often. PM performance was related to participants’ subjective total sleep time in a recent large-scale study (Kyle et al., 2017), with both long (> 9 h) and short (< 7 h) subjective sleep durations associated with worse PM performance. A smaller-scale study did not find a relationship between subjective sleep duration and PM (Scullin et al., 2019). In the studies we report here, we focused on the relationship of PM with subjective sleep measures, namely subjective sleepiness and subjective sleep quality.

### Prospective memory and subjective sleepiness

Subjective sleepiness is driven by poor and short sleep (e.g., Axelsson et al., 2008; Bonnet, 1985; Jewett, Dijk, Kronauer, & Dinges, 1999) in interaction with circadian rhythms (Borbély, 1982). It is associated with lowered levels of arousal (e.g., Santamaria & Chiappa, 1987) and manifests itself as a feeling of somnolence or drowsiness. The prevalence of elevated levels of sleepiness may reach up to 26% (for an overview, see Young, 2004). Thus, if subjective sleepiness is negatively related to PM, this may have widespread consequences.

In the only published study performed to investigate a possible relationship of subjective sleepiness with event-based PM, no such relationship was found (Leong, Koh, et al., 2019). However, because of its small sample size of 24 participants, this study does not warrant strong conclusions.

Studies from other cognitive domains suggest that there may be such a relationship and that sleepiness should primarily be associated with the prospective, not the retrospective component of PM. To derive this prediction, we must consider that the prospective component of nonfocal event-based PM tasks is based on attentional processes. Some studies have shown an association of stronger subjective sleepiness with worse sustained attention (Gillberg, Kecklund, & Åkerstedt, 1994; Gorgoni et al., 2014; Kaida, Åkerstedt, Kecklund, Nilsson, & Axelsson, 2007; Kaida et al., 2006). Therefore, subjective sleepiness should also be negatively associated with the prospective component of PM in our nonfocal PM task.

The retrospective component of the described PM task entails episodic recognition of PM targets. Therefore, if stronger subjective sleepiness was associated with worse recognition memory, it should also be negatively related to the retrospective component of PM. However, previous studies did not show a relationship between recognition memory and subjective sleepiness (Casey et al., 2016; Cousins, Sasmita, & Chee, 2018). The retrospective component of our PM task should, therefore, be unrelated to subjective sleepiness, and a possible relationship between sleepiness and PM performance should be due to its prospective, not its retrospective component.

### Prospective memory and subjective sleep quality

Poor subjective sleep quality is reported on at least one day per month in 69% of US citizens (Centers for Disease Control and Prevention, 2008). However, evidence regarding the relationship between PM and subjective sleep quality is scarce. There seems to be no correlation between subjective sleep quality and event-based PM performance in younger (Rendell, Gray, Henry, & Tolan, 2007; Rendell, Mazur, & Henry, 2009) or older adults (Cavuoto et al., 2016).

Although there does not seem to be a relationship between subjective sleep quality and PM performance, it might still be related to the prospective component of PM: Subjective sleep quality is positively related to sustained attention in younger (Benitez & Gunstad, 2012) and in older adults (Byun, Kim, & Riegel, 2017). This suggests that subjective sleep quality should be positively related to the prospective component of our nonfocal PM task, which relies on attentional processes.

In contrast, the retrospective component of PM should not be related to subjective sleep quality. The only study examining the relationship between young adults’ subjective sleep quality and retrospective recognition memory (Gobin, Banks, Fins, & Tartar, 2015) showed no association for neutral stimuli, which suggests that subjective sleep quality should not be related to the retrospective recognition component of our PM task.

### The present studies

The studies reported here were aimed at addressing two drawbacks in previous studies of the relationship between subjective sleepiness, subjective sleep quality, and PM. First, all these studies relied on PM hit rate (i.e., the proportion of PM targets correctly responded to). This is a common measure of PM performance, but it confounds the prospective and the retrospective components of PM, as both components contribute to performance. That is, in order to correctly respond to a PM target, participants have to remember that they must do something in addition to the ongoing task (prospective component) and must recognize the PM target (retrospective component). Separate measurement of the prospective and the retrospective component is, however, crucial for the purpose of the present studies: The literature suggests that if increased subjective sleepiness and low subjective sleep quality are associated with worse PM, this should be driven by deficits in the prospective, not the retrospective component. To investigate whether subjective sleepiness and sleep quality are related to the prospective component only, we used the multinomial processing tree (MPT) model of event-based PM (Smith & Bayen, 2004), which allowed us to obtain separate measures of the prospective and the retrospective components (the model is described in the Results section of Study 1).

The second drawback of the previous studies is their use of frequentist statistics. Frequentist statistics may be used to reject the null hypothesis, whereas Bayesian statistics also allow us to collect evidence in favor of the null hypothesis (e.g., Wagenmakers, Lee, Lodewyckx, & Iverson, 2008). Hence, using Bayesian statistics allowed us to directly compare evidence for the null vs. the alternative hypothesis. Thus, even if there is no association of subjective sleepiness or sleep quality with the prospective component of PM, we can quantify our evidence and assess the validity of the null hypothesis. In conclusion, as the Bayesian statistics used in the present studies render null results interpretable, these studies strengthen the argument for or against a relationship between subjective sleepiness or sleep quality and PM.

## Study 1

In Study 1, we investigated the relationship between subjective sleepiness and PM. Participants performed the computerized nonfocal PM task illustrated in Fig. 1. We expected possible relationships of subjective sleepiness and sleep quality with PM to be primarily driven by associations with the prospective component. We expected the prospective component to be negatively related to subjective sleepiness, as previous studies showed a negative relationship of sleepiness with sustained attention (e.g., Gillberg et al., 1994). Low subjective sleep quality may, if anything, be negatively related to the prospective component, but the small number of previous studies did not allow for clear predictions.

### Method

#### Participants

The Chair of the Research Ethics Committee of the College of Mathematics and Natural Sciences at the Heinrich-Heine-Universität Düsseldorf declared this study exempt from ethics review. To determine sample sizes, we used the same power considerations in both studies. An a priori power analysis showed that 104 participants were required to obtain a power of .80 to find a small-to-medium overall effect of *f*^2^ = .11 in a regression on PM hit rate with α = .05 and three predictors (subjective sleepiness before and after the study, and subjective sleep quality). We recruited on the university campus and via online social networks. For Study 1, the recruitment yielded 118 participants (87 female, 31 male; mean age = 21.42 years, *SD* = 3.02) who fulfilled the following inclusion criteria: German native speaker, between 17 and 30 years old, good mental and physical health, and did not report achromatopsia (because of the color-matching task). Additionally, they fulfilled the following inclusion criteria to ensure normal sleep behavior and normal cognitive functioning: (1) no sleep disorders, (2) no shift work in the past 3 months, (3) no more than four alcoholic beverages the night before, (4) not under the influence of pharmaceutical or recreational drugs that could impair attention, (5) non-smokers, as smoking has a detrimental effect on sleep (Cohrs et al., 2014; Jaehne et al., 2012), and (6) no previous participation in PM studies. Participants received monetary reimbursement or course credit. The data from all 118 participants entered the analyses, which resulted in a statistical power of .86.

#### Procedure, measures, and materials

Participants signed consent and were tested in groups of up to four in individual computer booths. The study was computer-based except for the paper-pencil questionnaires filled in at the end. At the beginning of the study, participants rated their current level of sleepiness on a computer-based version of the Karolinska Sleepiness Scale (KSS; Åkerstedt & Gillberg, 1990), which is a one-item 9-point Likert scale ranging from 1 (*extremely alert*) to 9 (*extremely sleepy – fighting sleep*). The KSS correlates with objective electroencephalogram (EEG) and electrooculogram (EOG) measures of sleepiness and is a valid tool for measuring sleepiness (Åkerstedt & Gillberg, 1990; Kaida et al., 2006). Then, the PM task was administered.

##### Ongoing color-matching task and prospective-memory task

As the ongoing task, we used the color-matching task (e.g., Smith & Bayen, 2004; see Fig. 1), in which participants indicated whether the color of a word matched that of one of four previously presented rectangles. Participants were instructed to press the *v* or *m* key to indicate whether or not the colors matched (with the assignment counterbalanced across participants). Rectangles and words were presented in red, blue, white, green, and yellow on a black background. The colors were presented equally often during the color-matching task. Color matches and non-matches occurred equally often and in random order. The words were written in Arial 24, and the rectangles were 120 × 166 pixels in size. Rectangles were presented for 500 ms each with interstimulus intervals of 250 ms. Words were presented until a response was made. Participants performed six practice ongoing-task trials.

Next, participants performed three blocks of a PM task embedded in the ongoing task (see Fig. 1). Three blocks were necessary to obtain a sufficient number of PM trials per person for MPT modeling without increasing the proportion of PM trials during the ongoing task (cf. Arnold, Bayen, & Böhm, 2015). Participants received instructions to perform a PM task in addition to the color-matching task. Specifically, they were instructed to press F1 whenever they encountered one of five previously studied PM target words. They were also told that if they accidentally performed the color-matching task on a PM target trial, they could still press the PM key during the rectangle presentation of the next trial. Then, five PM target words were presented in random order for 5 s each, black on white background in Arial 24.

We selected 165 words from the database by Lahl, Göritz, Pietrowsky, and Rosenberg (2009) for the color-matching and PM tasks. We divided the words into three lists of 55 words each. From each list, we chose five words as PM target words such that the lists as well as targets and distractors did not differ in valence, arousal, and concreteness (according to Lahl et al., 2009) as well as length, number of syllables, and frequency (according to the dlex database, Heister et al., 2011). One list was presented per block (in counterbalanced order), and each word was presented twice.

After presentation of the target words, participants had to solve simple addition problems of the form a + b + c for 3 min as a distractor task. Then, they performed 110 color-matching task trials, 10 of which included PM target words. Target trials occurred on every 8th to 12th trial (10th trial on average).

At the beginning of each of the three consecutive test blocks, the PM-task instructions were given, and participants studied five new PM target words followed by the 3-min distractor task. After completing all three blocks, participants indicated which key they were supposed to press upon encountering a PM target word. They were also asked whether they had remembered to press this key at any time during the study. Finally, participants filled in the computer-based KSS for a second time.

##### Paper-pencil questionnaires

Then, participants filled in a questionnaire to assess demographics and check the inclusion criteria. Next, they filled in a self-drafted questionnaire about caffeine consumption and the Fagerström-Test for nicotine dependency (Bleich, Havemann-Reinecke, & Kornhuber, 2002).^1^

Afterwards, they filled in the German version of the Pittsburgh Sleep Quality Index (PSQI; Buysse, Reynolds, Monk, Berman, & Kupfer, 1989; translated by Riemann & Backhaus, 1996), which captures subjective sleep quality during the previous 2 weeks in 10 questions. These can be subsumed in 7 components: (1) subjective sleep quality, (2) sleep latency, (3) sleep duration, (4) habitual sleep efficiency (i.e., the amount of time participants spent asleep in relation to the amount of time spent in bed), (5) sleep disturbances, (6) use of sleep medication, and (7) daytime dysfunctions. The component scores are summed to a global PSQI score that ranges from 0 to 21, with higher values indicating poorer sleep quality. To discriminate between good and bad sleepers, a cut-off value of > 6 has been suggested for the German version of the test (Backhaus, Junghanns, Broocks, Riemann, & Hohagen, 2002), resulting in a sensitivity of 93.4% and a specificity of 100%. Buysse et al. (1989) showed that the PSQI is valid, internally consistent, and highly reliable (Cronbach’s α = .83).

Next, participants filled in the morningness-eveningness questionnaire (Horne & Östberg, 1976) and a sleep diary for the previous night.^1^ Finally, the participants were debriefed and compensated.

### Results

We first report descriptive statistics of ongoing-task performance and PM-task performance. Then, to test whether subjective sleepiness and sleep quality were related to overall PM performance, we computed linear regressions on PM hit rate with PSQI^2^ and KSS scores as predictors. To test our predictions that subjective sleepiness and sleep quality should be related to the prospective component, we separated it from the retrospective component via MPT modeling using the Bayesian hierarchical latent-trait approach (Klauer, 2010). Then, we computed regressions on the prospective component with PSQI and KSS scores as predictors. Additional file 1 includes correlation tables for all measures reported in the manuscript.

For all analyses, we used Bayesian statistics to weigh the null and alternative hypotheses against each other. In Bayesian statistics, the Bayes factor (BF) indicates the likelihood with which the data are obtained under one hypothesis as compared to the other hypothesis. For instance, a *BF*_10_ of 3.2 indicates that the data are 3.2 times more likely under the alternative hypothesis as compared to the null hypothesis. Critically, the index of the *BF* indicates whether the null hypothesis is compared to the alternative hypothesis (01) or vice versa (10; e.g., Rouder, Morey, & Pratte, 2017). According to Raftery (1995, p. 139), a *BF* between 1 and 3 can be interpreted as “weak evidence,” a *BF* between 3 and 20 as “positive evidence,” a *BF* between 20 and 150 as “strong evidence,” and a *BF* larger than 150 as “very strong evidence” for one of the hypotheses. Except for the hierarchical MPT Bayesian analyses, we used JASP (Marsman & Wagenmakers, 2017) to conduct the Bayesian analyses in both studies. For each result, we report 95% Bayesian credibility intervals (BCIs, in brackets), which indicate the range in which the true value of the parameter lies with 95% confidence.

#### Ongoing-task performance

On average, participants answered correctly on 88% [87%, 89%] of the ongoing color-matching-task trials. Data and analyses regarding reaction times during the ongoing task are reported in Additional file 1.

#### Prospective-memory performance

We measured overall PM performance as the rate of PM target words correctly responded to (*PM hit rate*). If the PM key was pressed during presentation of the rectangles of the next trial following a PM target word, this was counted as a correct response (cf. Einstein & McDaniel, 1990).

**Fig. 2:**
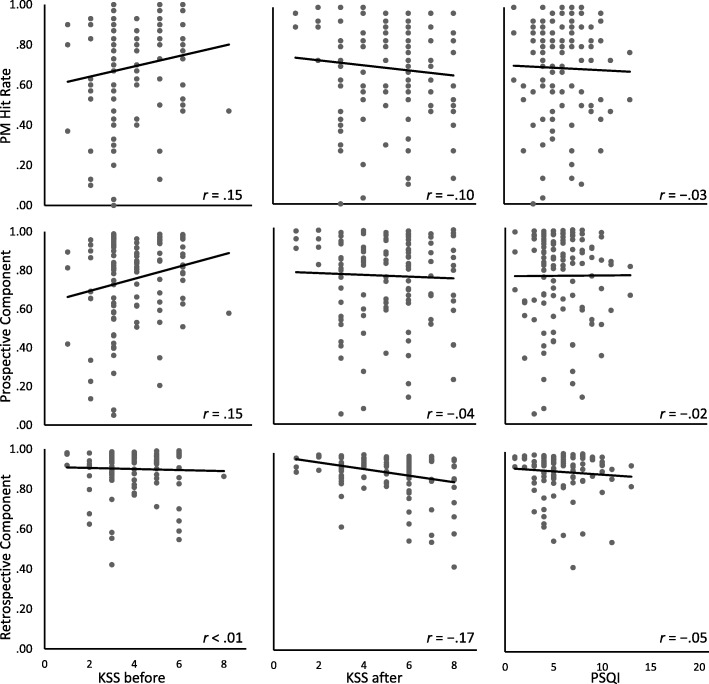
Scatterplots and correlations between the Karolinska Sleepiness Scale (KSS) ratings, the Pittsburgh Sleep Quality Index (PSQI), and the prospective-memory (PM) measures. Note that high KSS scores indicate strong subjective sleepiness, whereas high PSQI scores indicate low subjective sleep quality

We regressed PM hit rate (*Mean* = .69, [.65, .74]) on sleepiness ratings before and after the PM task^3^ (KSS_before_: *Mean* = 3.84, [3.59, 4.09]; KSS_after_: *Mean* = 5.09, [4.76, 5.43]), and on sleep quality (PSQI: *Mean* = 5.72, [5.27, 6.17]) by entering the predictors simultaneously. The predictors did not reliably explain any variance in PM hit rate, *R*^2^_korr_ = .04, *BF*_01_ = 2.89, with weak evidence for the null hypothesis. The scatterplots and regression lines in Fig. 2 illustrate the relationships of the PM hit rate, the prospective component, and the retrospective component with the PSQI and both KSS ratings.

#### Multinomial model of event-based prospective memory

To hit a PM target, participants must remember that they had to do something (prospective component) and must recognize the target word (retrospective component). A PM hit may also result from lucky guessing. PM hit rate is thus a conglomerate of several cognitive processes. As we expected subjective sleepiness and sleep quality to be primarily related to the prospective component of PM, it was important to separate this component from other cognitive processes. The MPT model of event-based PM introduced by Smith and Bayen (2004) provides an unconfounded measure by disentangling the components and thus enabled us to determine the associations of subjective sleepiness and sleep quality with the prospective component. In general, MPT models allow us to estimate the probabilities of latent processes underlying performance from frequencies of participant responses in a particular task. They are frequently used in cognitive psychology (for reviews, see Batchelder & Riefer, 1999, and Erdfelder et al., 2009). The MPT model of event-based PM was designed for ongoing tasks with two response options (e.g., the color-matching task where colors may or may not match) and an embedded PM task (e.g., when participants are required to press a specific key upon encountering target words). Thus, there are three response options: (a) color match, (b) no color match, and (c) the word is a PM target. There are four different trial types: (1) target, color match; (2) target, color non-match; (3) distractor, color match; (4) distractor, color non-match. As any of the three responses can be given on any of the four trial types, there are 12 different response categories. The response frequencies in each category are tallied and analyzed with the model.

**Fig. 3:**
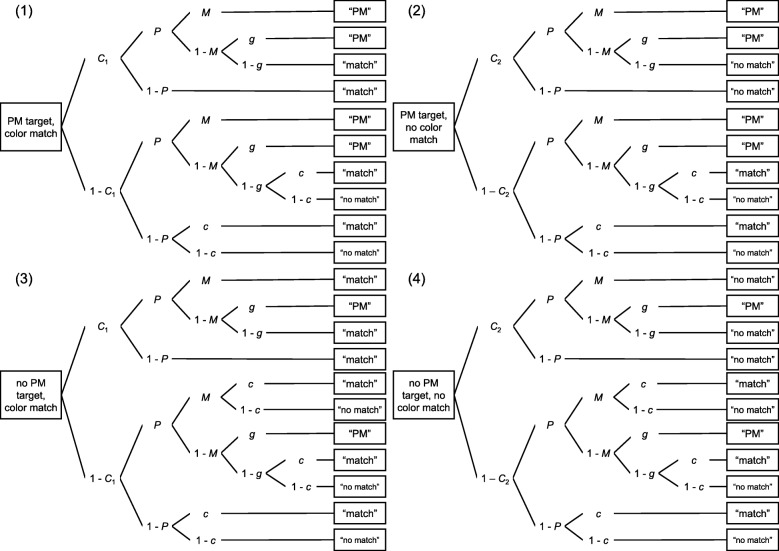
The multinomial processing tree model of event-based prospective memory by Smith and Bayen (2004). *C*_1_: probability to detect that the colors match; *C*_2_: probability to detect that the colors do not match; *P*: prospective component; *M:* retrospective component; *c*: probability to guess that the colors match; *g*: probability to guess that the word is a prospective-memory target. Adapted from “A multinomial model of event-based prospective memory” by R. E. Smith and U. J. Bayen, 2004, *Journal of Experimental Psychology: Learning, Memory, and Cognition, 30*, p. 758. Copyright 2004 by the American Psychological Association

The MPT model of event-based PM is shown in Fig. 3. Each of the trees represents one of the trial types. The first tree represents trials with a target word and a color match. Here, participants detect the color match with probability *C*_1_. They may further remember that they have an intention, with probability *P* (prospective component). With probability *M* (retrospective component), they may recognize the target word and thus push the PM key. If they do not recognize the target word, with probability 1 – *M*, they either guess that the word was a target (with probability *g*) or that it was a distractor (with probability 1 – *g*). If, on the other hand, participants do not remember that they have an intention, with probability 1 – *P*, they will answer “match” because they have detected the color match. The lower part of the tree represents cases in which participants do not detect the color match (with probability 1 – *C*_1_). Nevertheless, they may remember that they had an intention with a probability of *P*. If they recognize the target word (*M*), they answer “PM”. If alternatively, they do not recognize the word (1 – *M*), they guess that the word is a PM target (with probability *g*) or that it is not (1 – *g*). If they guess that the word is not a target, they must guess that colors match, with probability c, or do not match (1 – *c*) because they do not detect the color match. Similarly, if participants do not remember that they had an intention (with probability 1 – *P*), they guess that the colors match, with probability *c*, or do not match (1 – *c*).

The second tree refers to trials with a target word, but without a color match, and is constructed in a similar manner. In this tree, parameter *C*_2_ indicates the probability with which participants detect that colors do not match and leads to a “no match” response.

The third tree refers to trials without a target word and with a color match and is constructed in a similar manner as the first tree. With probability *M*, participants recognize the distractor as such. Whenever participants recognize the distractor or guess that the word is a distractor, they answer the color-matching task and do not press the PM key. Therefore, when participants do not detect the color match, but recognize the distractor as such, they guess that colors match (with probability *c*) or not (1 – *c*).

The fourth tree refers to trials without a target word and without a color match and is similar to the third tree. Here, parameter *C*_2_ represents the probability with which participants detect that the colors do not match.

Due to limited degrees of freedom, parameter restrictions need to be set to obtain an identifiable model. Following the recommendations by Smith and Bayen (2004), we set guessing parameter *c* to .50 (the actual ratio of color matches) and guessing parameter *g* to .09 (the actual ratio of target trials at test), assuming that guessing probabilities matched the actual ratios (*probability-matching*; e.g., Van Zandt, 2000).

The MPT model of PM has been shown to yield valid parameter estimates (Horn, Bayen, Smith, & Boywitt, 2011; Rummel, Boywitt, & Meiser, 2011; Smith & Bayen, 2004). It has been used in a number of previous studies to disentangle prospective and retrospective components of PM (Arnold & Bayen, 2019; Arnold, Bayen, & Böhm, 2015; Arnold, Bayen, & Smith, 2015; Pavawalla, Schmitter-Edgecombe, & Smith, 2012; Schnitzspahn, Horn, Bayen, & Kliegel, 2012; Smith & Bayen, 2005, 2006; Smith, Bayen, & Martin, 2010; Walter & Bayen, 2016).

The response frequencies obtained in Studies 1 and 2, summed over participants, are listed in Additional file 1. From the individual frequencies, we estimated model parameters for each participant for the prospective component (*P*), the retrospective component (*M*), and color-matching ability (*C*_1_ and *C*_2_) with the Bayesian hierarchical latent-trait approach (Klauer, 2010). The technical details have been explained elsewhere (Heck, Arnold, & Arnold, 2018; Klauer, 2010). This approach enabled us to compute regressions with subjective sleepiness and sleep quality explaining variance in individual parameters. Using this type of analysis, interactions between predictors cannot be taken into account, because setting a prior for such an interaction is an unresolved issue.

For the hierarchical MPT analyses, we used TreeBUGS (Heck et al., 2018), which uses Just Another Gibbs Sampler (JAGS, Plummer, 2013) to sample posterior distributions. We conducted 1,000,000 iterations, implemented a burn-in period of 500,000 samples, and retained every 100th sample. There was good convergence for every parameter, with the Gelman-Rubin statistic $\hat{R}$ < 1.05 (Gelman & Rubin, 1992).

#### Modeling results

Table 1 shows all group parameter estimates with 95% BCIs in brackets.

**Table 1:** Group parameter estimates for the MPT model and 95% Bayesian credibility intervals for Studies 1 and 2

Parameter	Study 1	Study 2	
	Upright	Upright	Supine
Prospective component *P*	.83 [.78, .89]	.81 [.73, .88]	.84 [.77, .91]
Retrospective component *M*	.94 [.92, .96]	.93 [.89, .96]	.87 [.82, .92]
Color match detection *C*_1_	.70 [.66, .73]	.58 [.47, .68]	.51 [.40, .63]
Color non-match detection *C*_2_	.87 [.85, .88]	.90 [.86, .92]	.87 [.83, .90]

$\hat{R}$ < 1.05 for all parameters; *MPT* multinomial processing tree

##### Prospective component

To test whether parameter *P* (the prospective component) was associated with subjective sleepiness and sleep quality, we conducted a regression analysis within the hierarchical MPT model described above. The KSS_before_ and KSS_after_ scores (as measures of subjective sleepiness) and the PSQI total score (as a measure of subjective sleep quality) were used to predict parameter *P*. Neither sleepiness ratings before, nor after the PM task, nor subjective sleep quality reliably predicted the prospective component *P*. Table 2 shows the regression weights, BCIs, and *BF*s_01_.^4^ All BFs indicated positive evidence for the null hypothesis; that is, with reasonable certainty, the sleep-related variables were not associated with the prospective component.

**Table 2:** Unstandardized regression weights, 95% Bayesian credibility intervals, and Bayes factors for all predictors in Study 1

PM component	Predictor	Regression weight	*BF*_01_
Prospective component *P*	KSS_before_	.13 [−.03, .30]	3.51
	KSS_after_	−.02 [−.14, .11]	10.75
	PSQI	−.01 [−.09, .07]	12.78
Retrospective component *M*	KSS_before_	.06 [−.08, .19]	14.94
	KSS_after_	−.08 [−.18, .01]	4.15
	PSQI	−.01 [−.08, .05]	12.16

*KSS* Karolinska Sleepiness Scale, *PSQI* Pittsburgh Sleep Quality Index, *BF*_01_ Bayes factor indicating evidence for the null hypothesis

##### Retrospective component

Similarly, we repeated these analyses for parameter *M* (retrospective component). All BFs indicated positive evidence that neither sleepiness ratings before, nor after the PM task, nor subjective sleep quality predicted parameter *M*. This aligns with other studies that did not find a relationship between subjective sleepiness and recognition memory (e.g., Casey et al., 2016). Additional analyses regarding ongoing-task parameters *C*_1_ and *C*_2_ are provided in Additional file 1.

### Discussion

All BFs indicated positive evidence for the null hypotheses; that is, subjective sleepiness and sleep quality were neither associated with PM hit rate nor with the PM components. These results were unexpected for the prospective component, because previous studies on sustained attention (e.g., Gillberg et al., 1994) suggested that subjective sleepiness might be negatively associated with the prospective component of PM. Subjective sleep quality did not predict PM hit rate, which supports findings from other PM studies (e.g., Rendell et al., 2007). However, this result is not in line with studies showing an association between subjective sleep quality and attention (e.g., Benitez & Gunstad, 2012).

Correlational studies such as Study 1 and most prior studies of relationships between subjective sleepiness and cognitive variables must be interpreted with some caution, as they are based on correlations, thus not allowing conclusions about directional relationships. On the one hand, subjective sleepiness may lead to worse PM. However, the causal relationship may also be reversed. That is, participants who are aware of their own poor PM performance might attribute it to sleepiness, which would lead to lower sleepiness ratings in participants with worse PM. In Study 2, we therefore aimed to experimentally induce subjective sleepiness.

## Study 2

In Study 2, we aimed to corroborate the results from Study 1 by using an experimental manipulation. We chose not to sleep deprive participants, because sleep deprivation leads to extreme levels of subjective sleepiness (e.g., Van Dongen, Maislin, Mullington, & Dinges, 2003) and elevated levels of cortisol (e.g., Minkel et al., 2014). Thus, sleep deprivation does not reflect the high levels of subjective sleepiness commonly found in everyday life, but it induces extreme sleepiness and thereby elicits stress responses that may confound the relationship between sleepiness and PM. Instead, we induced sleepiness by manipulating body posture. Lying in a supine posture increases sleepiness in comparison with sitting upright (e.g., Cole, 1989; Kräuchi, Cajochen, & Wirz-Justice, 1997). Therefore, in Study 2, we had participants perform the task in either upright or supine posture.

The mechanisms underlying the sleepiness-inducing effect of supine posture are of a physiological nature (i.e., changes in body temperature and blood pressure; cf. Caldwell, Prazinko, & Caldwell, 2003). One presumed mechanism is that when people lie down, activity in baroreceptors (i.e., receptors that capture blood pressure) is increased as a response to an increase in systolic blood pressure. The increased firing of these baroreceptors in supine posture in turn leads to decreased EEG arousal, thereby increasing both subjective and objective sleepiness. Thus, in supine posture, humans experience less physiological arousal than in upright posture.

Lowered levels of arousal induced by body-posture manipulations are associated with worse sustained attention (Caldwell et al., 2003), which is why our predictions primarily concerned the prospective component of PM in our nonfocal PM task. We deemed two outcomes possible given previous results. The first is that additional, posture-induced sleepiness does not affect the prospective component, as we found no relationship between sleepiness and the prospective component in Study 1. In this case, Study 2 should show no difference between postures. The alternative is that supine posture elicits stronger sleepiness affecting attentional processes, thus impairing the prospective component. In this case, the prospective component should be lower in supine than in upright posture.

Subjective sleepiness or poor sleep quality alone may not impair PM, but some studies indicated that sleep-related variables may interact to impair PM. Fabbri et al. (2014) found an effect of sleep quality on PM in an extreme-group comparison, where both groups differed on several sleep-related variables. Additionally, Fabbri, Tonetti, Martoni, and Natale (2015) showed that sleep quality was related to PM in patients with insomnia who suffered from severe daytime sleepiness. Thus, PM may be impaired when participants feel sleepy *and* report poor sleep quality. The manipulation of posture enabled us to determine whether posture-induced sleepiness and sleep quality have a combined effect on PM components. Furthermore, Muehlhan, Marxen, Landsiedel, Malberg, and Zaunseder (2014) showed a combined effect of posture-induced sleepiness and subjective sleep quality on reaction times in a working-memory task. As the prospective component of nonfocal PM tasks is associated with working memory (Arnold, Bayen, & Smith, 2015; Smith & Bayen, 2005), we expected a similar pattern of results for the prospective component of PM as the pattern shown by Muehlhan et al. (2014) for working memory. That is, we expected posture to moderate the relationship between subjective sleep quality and the prospective component. Specifically, a positive relationship between subjective sleep quality and the prospective component should be stronger under higher levels of sleepiness induced by supine posture.

### Method

#### Design and participants

The power considerations were the same as for Study 1. In Study 2, the three predictors on which we regressed PM hit rate were body posture, sleep quality, and the interaction of body posture and sleep quality.

Of a total of 105 students (81 female, 24 male; mean age = 22.36 years, *SD* = 2.91), 52 were randomly assigned to the upright group and 53 to the supine group. Participant recruitment and compensation were the same as in Study 1. Inclusion criteria were the same as in Study 1 with two additional criteria: (1) Individuals with visual impairment could only participate with a correction device that allowed flawless presentation via the head-mounted display used in Study 2 (i.e., contact lenses were allowed, but glasses were not); and (2) participants did not suffer from clinically relevant daytime sleepiness (i.e., Epworth Sleepiness Scale [ESS] total score < 11; Johns, 2000), as we wanted to investigate normal levels of sleepiness.

#### Procedure, measures, and materials

Prior to Study 2, participants completed an online screening questionnaire inquiring the inclusion criteria. If they fulfilled all criteria, they could sign up to participate. Participants signed consent and were tested individually. To double-check for inclusion criteria, participants were administered the Ishihara test of color vision (Ishihara, 1972), a demographic questionnaire, and several sleep-related paper-pencil questionnaires in the following order: the KSS, the ESS (Johns, 1991; translated by Bloch, Schoch, Zhang, & Russi, 1999), and the PSQI. The ESS consists of eight items that regard the probability to fall asleep in certain situations (e.g., while watching TV) on a 4-point Likert scale from 0 to 3. High total sum scores indicate high levels of daytime sleepiness.

##### Experimental set-up and technical equipment

During the computer-based study, the upright group sat in a chair, while the supine group lay down face up on a bed free of covers or pillows. Aside from that, the procedure was the same in both groups. An RB-740 response pad (Cedrus Corporation) with one horizontal row of seven keys was placed in the participant’s lap. The index fingers were placed on the 3rd and 5th keys, which were needed for the following tasks. All participants wore a Sony HMZ-T2 head-mounted display, which had a resolution of 1920 × 1080 pixels and two light-emitting diode displays, one for each eye. The head-mounted display was used to standardize the distance between eyes and screen for both groups and was adjusted until the participant could clearly read the presented text. Only indirect, dim light was allowed in the room to avoid light reflections on the lenses. Participants were asked to read a short text aloud to verify that they could see information presented on the display. Then, the study started.

##### Ongoing color-matching task and prospective-memory task

We made small changes to the procedure and materials of the color-matching task and the PM task used in Study 1, because in Study 2, we used a response pad instead of a keyboard. The words were presented in Arial 28 to facilitate the perception of blue words presented on a black background via the head-mounted display. As the distractor task, participants indicated via key press whether simple addition problems of the form a + b + c = d were correct or not. The middle (4th) key on the response pad served as the PM key. For the ongoing task and the distractor task, participants pressed the keys to the left and right of the PM key (i.e., the 3rd and 5th keys).

##### Psychomotor vigilance task

For the next 10 min, participants performed the psychomotor vigilance task (PVT; Dinges & Powell, 1985), which is commonly used to measure sustained attention under sleep deprivation and severe sleepiness (Van Dongen et al., 2003). Participants monitored a white screen and were required to press the middle key on the response pad as fast as possible whenever a black counter (in Arial 24) appeared. Upon key press, the counter stopped and showed the reaction time in milliseconds. The counter appeared every 2 to 10 s at random intervals. If the participants did not react for 10 s, the words “no reaction” appeared on screen for 1 s. If the participants pressed the middle key although the counter was not present, the words “false alarm” appeared for 1 s.

Afterwards, the participants removed the head-mounted display, and filled in the KSS for a second time. Finally, they were debriefed and compensated.

### Results

We present results in the following order. We first determined the success of the randomization. We then determined the effects of the experimental manipulation of posture on sleepiness followed by a comparison of both posture groups regarding ongoing-task and PM performance. Afterwards, we obtained an unconfounded measure of the prospective component of PM via MPT modeling using the Bayesian hierarchical latent-trait approach (Klauer, 2010) as in Study 1. We compared both posture groups regarding the prospective and the retrospective components. In addition, we performed moderator analyses to investigate whether the relationship between sleep quality and the prospective component depended on body posture. Refer to Additional file 1 for correlation tables for all measures reported.

#### Randomization check

Table 3 shows the descriptive data for randomization checks. At study outset, the two groups did not differ regarding daytime sleepiness as measured with the ESS, *BF*_01_ = 2.95, indicating successful randomization. The groups also did not differ regarding sleep quality, *BF*_01_ = 1.31. The mean PSQI scores of the supine group and the upright group were lower than the cut-off value of 6 for poor sleep quality, supine: *BF*_10_ = 678.30; upright: *BF*_10_ = 561,505. Thus, both groups can be characterized as good sleepers.

**Table 3:** Means and 95% Bayesian credibility intervals (in brackets) of measures obtained with the Epworth Sleepiness Scale, the psychomotor vigilance task, and the Karolinska Sleepiness Scale for the two posture groups in Study 2

Measure	Upright	Supine
ESS	5.50 [4.86, 6.14]	6.00 [5.30, 6.70]
PSQI	4.15 [3.58, 4.73]	4.83 [4.29, 5.37]
PVT		
False alarms	3.35 [1.58, 5.11]	2.64 [1.18, 4.10]
Lapses	0.00	0.04 [−0.02, 0.09]
Logarithmized reaction times	5.79 [5.74, 5.83]	5.82 [5.79, 5.85]
KSS		
Time 1	3.32 [2.97, 3.67]	3.25 [2.92, 3.57]
Time 2	4.12 [3.62, 4.61]	4.62 [4.18, 5.07]

*ESS* Epworth Sleepiness Scale, *PSQI* Pittsburgh Sleep Quality Index, *PVT* psychomotor vigilance task, *KSS* Karolinska Sleepiness Scale

#### Effects of posture on sleepiness and vigilance

Table 3 shows the descriptive data obtained with the KSS and the PVT. We analyzed whether supine posture resulted in increased subjective sleepiness as measured with the KSS. A Bayesian analysis of variance (ANOVA)^5^ with posture (upright vs. supine) as between-subjects factor and time of measurement (before vs. after the study) as within-subjects factor yielded no main effect of posture, *BF*_Inclusion_^6^ = 0.40, but a main effect of time, *BF*_Inclusion_ = 1.96*10^7^, and an interaction, *BF*_Inclusion_ = 2.41. Participants felt less sleepy before the session than afterwards. As expected, this increase was more pronounced in the supine group. Supine posture thus induced subjective sleepiness.

Then, we analyzed the effects of posture on vigilance as measured with the PVT, specifically lapses, false alarms, and logarithmized reaction times.^7^ The BF indicated no group differences regarding false alarms, *BF*_01_ = 4.09, and logarithmized reaction times, *BF*_01_ = 2.67, but it could not be computed for lapses due to their rare occurrence (two in the supine-posture group overall). Supine posture thus induced sleepiness when measured by the subjective KSS, but this effect did not show in the behavioral PVT measures.

#### Ongoing-task performance

The posture groups did not differ in rate of correct responses in the color-matching task (*Mean*_upright_ = .86, [.84, .89], *Mean*_supine_ = .84, [.81, .87]), *BF*_01_ = 3.01. Refer to Additional file 1 for additional analyses of reaction times in the color-matching task.

#### Prospective-memory performance

**Fig. 4:**
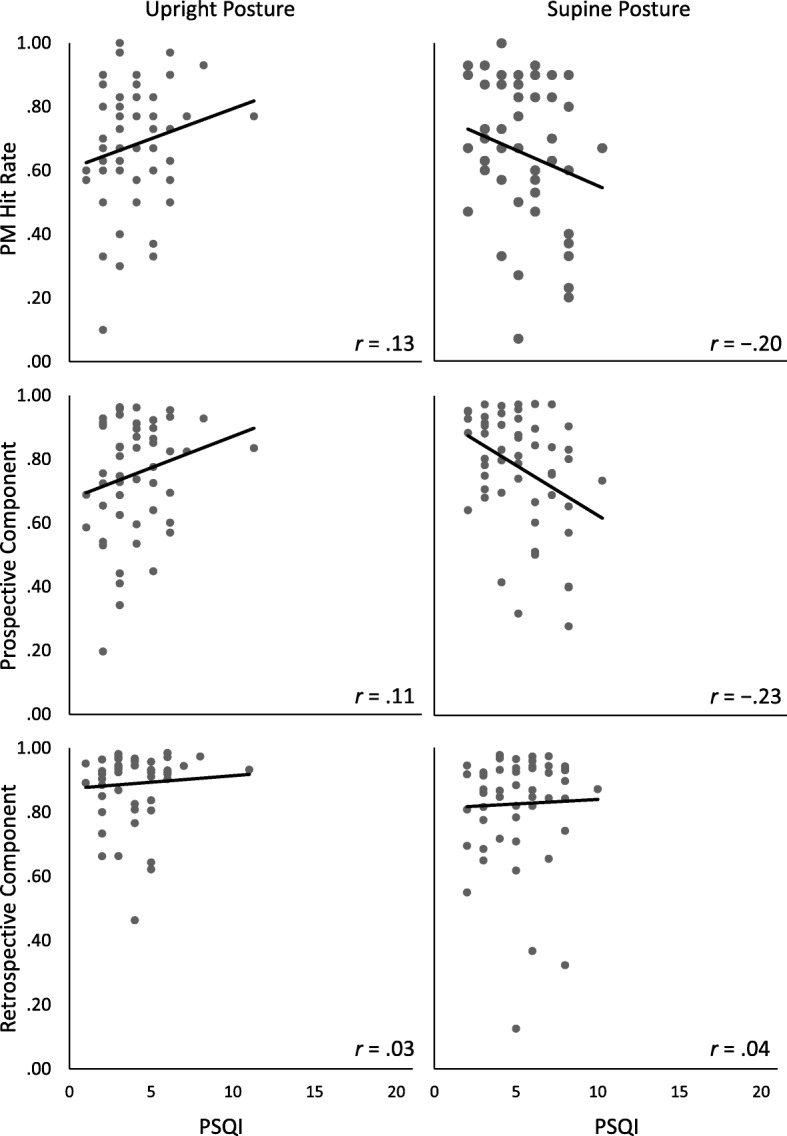
Scatterplots of the correlations between the Pittsburgh Sleep Quality Index (PSQI) and the prospective-memory (PM) measures for each posture group. Note that higher scores on the PSQI indicate poorer subjective sleep quality

The posture groups did not differ in PM hit rate (*Mean*_upright_ = .68, [.63, .73]; *Mean*_supine_ = .66, [.60, .72]), *BF*_01_ = 4.26. To test the moderator hypothesis that subjective sleep quality correlated with PM hit rate in the supine group only, we computed a regression to predict PM hit rate. The predictors were posture (supine vs. upright), PSQI score, and the interaction between posture and PSQI.^2^ If the interaction was a significant predictor of PM hit rate, this would indicate that the relationship between PM hit rate and subjective sleep quality varied between posture groups (i.e., posture would moderate this relationship). However, the regression model did not explain variance in PM hit rate, *R*^2^_corr_ < .01, *BF*_01_ = 10.34. Thus, the relationship between PM hit rate and sleep quality did not differ depending on posture. The scatterplots and regression lines in Fig. 4 illustrate the relationships of PM hit rate, the prospective component, and the retrospective component with the PSQI.

#### Modeling results

We estimated parameters for each group separately. Table 1 shows the group parameter estimates and 95% BCIs. We used the PSQI total score to predict parameters *P* (and, additionally, *M*) in both posture groups separately. The settings for the estimation process were identical to those in Study 1.

##### Prospective component

The posture groups did not differ regarding the prospective component *P*, as indicated by the 95% BCI of the parameter difference, which included zero, Δ*P* = − .03 [−.13, .07]. To examine moderator effects of body posture on the relationship between sleep quality and the prospective component, we estimated regression slopes between the PSQI and parameter *P*, separately for each posture group, and compared these slopes between groups.^2^ In the upright group, the regression weight of sleep quality predicting the prospective component *P* did not differ from zero, *b* = .08 [−.06, .23], *BF*_01_ = 5.34, with the BF indicating positive evidence for the null hypothesis, that is, no relationship between sleep quality and the prospective component *P*. In the supine group, the regression weight of sleep quality predicting the prospective component *P* was not different from zero either, *b* = −.13 [−.27, .01], *BF*_01_ = 1.62, with the BF favoring the null hypothesis, albeit yielding only weak evidence. However, these regression weights differed from each other, Δ*b* = .20, [.01, .40]. This means that sleep quality was associated with the prospective component in different ways, depending on posture. Although the regression weights were not different from zero, the weight tended to be negative in the supine-posture group and close to zero/positive in the upright group. In supine posture, participants with better sleep quality (i.e., lower values on the PSQI) tended to have a higher probability to remember that they needed to fulfill an intention. In the upright group, sleep quality was not associated with the probability to remember having an intention.

##### Retrospective component

We repeated these analyses for the retrospective component *M*. The two groups did not differ regarding parameter *M*, Δ*M* = .06 [−.004, .12]. This adds to the studies that found no effects of sleepiness on recognition memory (e.g., Study 1 above; Casey et al., 2016). The regression weight of sleep quality predicting parameter *M* was neither different from zero in the upright group, *b* = .03 [−.10, .15], *BF*_01_ = 9.54, nor in the supine group, *b* = .04 [−.08, .15], *BF*_01_ = 8.90, with both BFs yielding positive evidence for the null hypothesis.

##### Ongoing-task parameters

The posture groups did not differ regarding color-matching parameters *C*_1_ and *C*_2_, Δ*C*_1_ = .06 [−.09, .22], Δ*C*_2_ = .03 [−.02, .07]. Thus, the lack of group differences in the PM components cannot be explained by a trade-off between the PM task and the ongoing task. Refer to Additional file 1 for additional analyses regarding parameters *C*_1_ and *C*_2_.

Overall, the posture groups did not differ regarding either component of PM. However, the relationship between subjective sleep quality and the prospective component *P* varied depending on posture. Thus, body posture was a moderator of this relationship.

### Discussion

In Study 2, we aimed to determine effects of sleepiness induced by supine posture on PM and placed participants in upright or supine posture during a nonfocal event-based PM task. Supine posture induced subjective sleepiness. In upright posture, the BFs again showed convincing evidence against an association of sleepiness and subjective sleep quality with PM and its components. The posture groups did not differ regarding the PM components. However, posture moderated the relationship between subjective sleep quality and the prospective component: In supine posture, poor subjective sleep quality was more negatively related to the prospective component compared to the association in upright posture.

The PVT as a measure of sustained attention was unaffected by posture. The lack of effects of posture on sustained attention may explain why posture did not impair the prospective component: The prospective component should only be affected if the underlying attentional processes suffered from posture-induced sleepiness.

The body-posture manipulation successfully induced subjective sleepiness. Further, this manipulation resulted in moderate levels of subjective sleepiness which are common in everyday life, unlike sleep-deprivation or sleep-restriction protocols which rather induce extreme levels of sleepiness (e.g., Grundgeiger et al., 2014, who found a mean KSS score of 7.89 after a night of total sleep deprivation). Manipulating body posture is a relatively simple way to investigate moderate levels of subjective sleepiness while avoiding strong stress responses such as those resulting after sleep deprivation (e.g., Minkel et al., 2014). However, corroborating the results from Study 1, the body-posture manipulation did not relate to the behavioral performance measure.

## General discussion

We conducted two studies to investigate the association of subjective sleepiness and sleep quality with PM and its components. These studies make a compelling case for Bayesian statistics in handling null effects and forestalling publication bias: Study 1 yielded positive evidence for the null hypothesis that subjective sleepiness and sleep quality would not be associated with PM components. Study 2 showed no difference between upright and sleepiness-inducing supine posture in PM and its components, and showed a moderation: The relationship between subjective sleep quality and the prospective component differed depending on posture. We discuss theoretical aspects, and then applied implications.

In neither study was sleepiness related to the prospective component of PM as measured by the MPT model, which contrasts with previous studies on attention (e.g., Gillberg et al., 1994). We deliberately investigated moderate sleepiness, as common in everyday life, to maximize ecological validity. Our results complement the study by Leong, Koh, et al. (2019), who did not find correlations between PM and subjective sleepiness, but who used a small sample size and an overall performance measure only.

Subjective sleep quality was also not associated with the prospective component of PM in either study. This is in line with studies investigating subjective sleep quality and PM (e.g., Rendell et al., 2007) which, however, also used an overall performance measure only. As the prospective component in nonfocal PM tasks is based on attentional processes, our findings are in line with evidence against effects of subjective sleep quality on attention (e.g., Benitez & Gunstad, 2012).

Compensation may explain why neither sleepiness nor subjective sleep quality alone was associated with the prospective component. Drummond, Gillin, and Brown (2001) showed that activation in brain regions related to attention is positively related to subjective sleepiness after sleep deprivation, thereby reducing impairments in attention-switching tasks. The same may hold for PM tasks, leading to a preserved prospective component when one is sleepy.

Body posture did however moderate the relationship between subjective sleep quality and the prospective component in Study 2, which concurs with findings from PM research (Fabbri et al., 2014, 2015) and working-memory research (Muehlhan et al., 2014). The moderation suggests that the association of subjective sleep quality with the prospective component depends on whether task conditions promote sleepiness, such as supine posture does.

The moderating effect of body posture on the relationship between subjective sleep quality and the prospective component of PM may be explained by differences in arousal levels between both groups. As outlined in the introduction to Study 2, the physiological mechanisms of the body-posture manipulation lead to lower arousal in supine posture than upright posture. We propose that under sleepiness-promoting conditions of low arousal (e.g., in supine posture), the prospective component is more strongly associated with subjective sleep quality, because participants fail to compensate when feeling sleepy and having poor subjective sleep quality.

Regarding the retrospective component, as measured by the MPT model, both studies yielded evidence for the null hypothesis. That is, neither sleepiness nor subjective sleep quality were related to this component. This adds to the literature speaking against an effect of sleepiness and sleep quality on recognition (e.g., Casey et al., 2016). The retrospective component is based on recognition in our studies and may be spared by subjective sleepiness and sleep quality, because recognition largely relies on automatic familiarity (Mandler, 1980). In fact, Swann, Yelland, Redman, and Rajaratnam (2006) showed stronger reliance on automatic processes after inducing subjective sleepiness via sleep restriction, which preserved recognition memory. However, as apparent from the scatterplots in Figs. 2 and 4, the parameter estimates for the retrospective component were fairly high. Thus, a ceiling effect may have masked correlations with this parameter.

Although our results might suggest that subjective sleepiness was not associated with PM, this does not mean that sleep-related variables do not impact PM at all. One needs to bear in mind that we only investigated moderate levels of subjective sleepiness that did not show in sustained attention in Study 2. It is reassuring to know that moderate levels of sleepiness or poor subjective sleep quality by themselves do not compromise PM, as short sleep and complaints about poor sleep quality are increasing in prevalence (e.g., Kronholm et al., 2008). In contrast, extreme levels of sleepiness, such as after sleep deprivation or sleep restriction, may compromise PM (e.g., Grundgeiger et al., 2014). Also, in patients with sleep disorders who often suffer from deficiencies in several sleep-related variables, PM impairment may be strong (e.g., Bezdicek et al., 2018).

Levels of subjective sleepiness were increased after the PM task in both studies. A possible reason may be that in the KSS ratings, subjective sleepiness may have been confounded with acute fatigue. Acute fatigue and sleepiness are two related, yet different constructs. Acute fatigue results from exhausting, sometimes cognitive, effort and may be alleviated by wakeful rest (cf. Shen, Barbera, & Shapiro, 2006). In the present studies, fatigue may have arisen from completing more than 300 trials of the ongoing color-matching task, while additionally keeping the PM task in mind. In contrast, subjective sleepiness describes the subjective feelings experienced during the long-running, slow transition from a state of alert wakefulness to a state of sleep. It results from the interaction of prolonged wakefulness with circadian rhythms (Borbély, 1982). To our knowledge, there are no published studies directly investigating PM and fatigue. However, this discussion may be informed by research on depletion and PM, as depletion may be reflected in higher levels of fatigue (e.g., Lim et al., 2010). Several studies (Cook, Ball, & Brewer, 2014; Shelton et al., 2013) showed that depletion before a nonfocal PM task did not impact PM performance. However, levels of *subjective* fatigue were correlated with PM performance (Cook et al., 2014). This suggests that if the KSS scores in the present study were influenced by fatigue, we might have found a correlation between PM and the KSS measure—which we did not. Although we cannot with certainty exclude a confound of the KSS with fatigue, we deem this unlikely based on these findings.

One limitation of the present study regards the small range in PSQI scores, because we chose to include healthy participants only. We did so to avoid sleep disorders as a possible confound. However, limiting the sample like this also led to a small range in the PSQI, which may have forestalled finding correlations between the PSQI and PM measures. Regarding the KSS, by contrast, the scatterplots in Fig. 2 nicely show that almost the whole range of possible ratings was covered. This indicates that even natural variability in sleepiness was substantial and that restriction of range was not an issue for this variable.

Another possible concern regarding the null correlations between PM components and sleep-related variables might be the lower number of participants per group in Study 2. However, the Bayes factors in both studies indicated that we collected sufficient evidence (i.e., *BF* > 3) in favor of the null hypotheses. Moreover, Study 1 had a larger sample size, yet yielded the same results as Study 2. Thus, we think that sample size is not a concern for our findings.

Our findings regarding supine posture may be of practical importance for the workplace use of dynamic workstations, which allow switching from standing, to sitting, to reclined postures (US Patent No. 8,939,500, Voigt & Speicher, 2015). The possibility to lie down at work thereby temporarily alleviating back pain (Waddell, 1992) and preventing chronic back problems may reduce absenteeism, economic cost, and mental-health problems related to pain (e.g., Hagen, Svensen, Eriksen, Ihlebaek, & Ursin, 2006). In addition, prolonged sitting is related to increased mortality rates (Chau et al., 2013). Our findings imply that dynamic workstations likewise do not influence work-related memory function.

The findings of Study 2 also have implications for functional magnetic resonance imaging (fMRI) studies. Researchers conducting fMRI may consider taking participants’ subjective sleep quality into account, because it may be related to the results obtained from participants lying in a scanner in supine position. Particular caution should be applied in fMRI studies when comparing healthy controls and participants with health issues that affect subjective sleep quality, e.g., depression (O'Leary, Small, Panaite, Bylsma, & Rottenberg, 2017).

Overall, the present studies show that the prospective and retrospective components of PM are unrelated to subjective sleepiness and sleep quality and underline the need to study subjective measures of sleep, as the results may deviate from those obtained with objective sleep measures (e.g., Scullin & McDaniel, 2010). An interesting direction for future research would be to directly compare effects of subjective and objective sleep-related variables on components of PM.

## Supplementary information

**Additional file 1.** Ongoing-task measures, response frequencies, frequentist analyses, and correlation matrices.

## Data Availability

The datasets supporting the conclusions of this article are available in the Open Science Framework repository, https://osf.io/z3yk7/.

## Abbreviations

BCI: Bayesian credibility interval
BF: Bayes factor
EEG: Electroencephalogram
ESS: Epworth Sleepiness Scale
EOG: Electrooculogram
fMRI: Functional magnetic resonance imaging
KSS: Karolinska Sleepiness Scale
MPT: Multinomial processing tree
PM: Prospective memory
PSQI: Pittsburgh Sleep Quality Index
PVT: Psychomotor vigilance task

## Footnotes

1 Caffeine consumption, morningness-eveningness, and variables derived from the sleep diary did not correlate with PM and are not reported in this paper.

2 Using the PSQI component of “subjective sleep quality” (component 1) as a predictor did not change the pattern of results for any of the analyses reported in either study.

3 One may argue that a combined KSS score (e.g., the sum of the before and after scores) is a more appropriate proxy of sleepiness during the PM task. We reran all reported regression analyses in Study 1 using the KSS sum scores instead of the before and after scores as predictor. This yielded the same pattern of results: The KSS sum score was not related to PM or any component, all *BF*s_01_ > 6.62.

4 The BFs for the regression weights were computed from analyses that included only one predictor at a time.

5 Two participants had to be excluded from this analysis due to missing data in the KSS.

6 For Bayesian repeated-measures ANOVAs, the *BF*_10_ and *BF*_01_ indicate the likelihood of the whole model, not that of the main effects and interactions separately. The *BF*_Inclusion_ reported here represents the Bayes factor for the main effects and the interactions separately.

7 The pattern of results was the same for non-logarithmized reaction times.
