# Supplementary material for: Are subjective sleepiness and sleep quality related to prospective memory?
Source: Cogn Res Princ Implic. 2020 Feb 7;5:5. doi: 10.1186/s41235-019-0199-7 (PMC7007451; doi:10.1186/s41235-019-0199-7)
Supplement: Supplementary file 1 — Additional file 1. Ongoing-task measures, response frequencies, frequentist analyses, and correlation matrices. [file 41235_2019_199_MOESM1_ESM.docx]

Are Subjective Sleepiness and Sleep Quality Related to Prospective Memory?

Additional File 1

Mateja F. Böhm, Ute J. Bayen, and Marie Luisa Schaper

Heinrich-Heine-Universität Düsseldorf

**Supplement 1: Effects of sleepiness and sleep quality on ongoing-task measures**

As reported in the article, sleepiness and sleep quality did not predict prospective memory (PM) and its components. One may hypothesize that this was the case because of a trade-off between the PM task and the ongoing task (OT). That is, participants with high levels of sleepiness or poor sleep quality may have compensated for their impairments by allocating attention away from the OT and toward the PM task. As a result, sleepiness and sleep quality would not affect the PM task, but slow reaction times (RTs) on the OT, and possibly decrease OT performance and the OT parameters *C*_1_ and *C*_2_ of the multinomial processing-tree (MPT) model. To test these possibilities, we performed supplemental analyses on RTs in the OT, OT performance, and OT model parameters, and report these in this additional file.

**Reaction Times in the Ongoing Color-Matching Task**

To investigate whether sleepiness and sleep quality slowed RTs, we conducted supplemental analyses in which the Karolinska Sleepiness Scale (KSS; as measure of sleepiness) and the Pittsburgh Sleep Quality Index (PSQI; as measure of sleep quality) served as predictors of OT RTs (Studies 1 and 2), and we compared OT RTs between posture groups (Study 2).

**Study 1.** To analyze OT RTs, we excluded RTs under 300 ms and longer than two standard deviations from each individual’s mean (cf. Rummel & Meiser, 2013). Furthermore, we only included correct ongoing-task trials. We excluded PM target trials and PM false alarms as well as the first trial following each PM target trial and false alarm. Mean RT was 1,270 ms (95% CI [1,196, 1,343]). Because the values differed significantly from a normal distribution, *W* = .92, *p* < .001, we logarithmized RTs, after which they did not significantly differ from a normal distribution, *W* = .99, *p* = .518. Neither sleepiness before and after the PM task nor sleep-quality ratings predicted the logarithmized RTs in the OT. Test statistics for this and all following regression analyses are reported in Table S1.

**Study 2.** We removed RT outliers as in Study 1 and included the same type of trials in our analyses. Again, mean OT RTs differed significantly from a normal distribution, *W* = .96, *p* = .002, but logarithmized RTs did not, *W* = .99, *p* = .578. Mean RT was 1,240 ms (95% CI [1,153, 1,327]) in the upright-posture group and 1,301 ms (95% CI [1,206, 1,395]) in the supine-posture group. The two posture groups did not differ regarding logarithmized RTs, *t*(103) = .93, *p* = .355, *d* = .18, *BF*_01_ = 3.30. A linear regression showed that posture (supine vs. upright), PSQI score, and the interaction of posture and PSQI did not predict logarithmized RTs.

Overall, the results of both experiments indicate that sleepiness and sleep quality did not slow RTs on the OT. Thus, the RT analyses did not support the hypothesis that the absence of an effect of sleepiness and sleep quality on PM was due to allocation of attention away from the OT and toward the PM task.

**Ongoing-Task Performance and Ongoing-Task Parameters *C*_1_ and *C*_2_**

To investigate whether sleepiness and sleep quality affected performance on the OT (measured as percent correct) and the MPT model parameters for the OT, *C*_1_ and *C*_2_, we conducted supplemental analyses in which KSS and PSQI served as predictors of OT performance and OT parameters (Studies 1 and 2), and we compared OT performance and OT parameters between posture groups (Study 2).

**Study 1.** Neither the KSS before and after the PM task, nor the PSQI predicted OT performance. Furthermore, neither the KSS before and after the PM task, nor the PSQI predicted parameter *C*_1_ or parameter *C*_2_.

**Study 2.** Posture, PSQI, and the interaction of posture and PSQI did not predict OT performance (i.e., proportion correct). Bayesian hierarchical MPT modeling indicated that the posture groups differed neither in parameter *C*_1_, ∆*C*_1_ = .06, 95% BCI [-.09, .22], nor in parameter *C*_2_, ∆*C*_2_ = .03, 95% BCI [-.02, .07]. Furthermore, in neither posture group did the PSQI predict parameter *C*_1_ or parameter *C*_2_. The regression weights of the PSQI predicting parameter *C*_1_ did not differ between posture groups, ∆*b* = .06, 95% BCI [-.11, .22]. The same was true for the regression weights of the PSQI predicting parameter *C*_2_, ∆*b* = -.05, 95% BCI [-.14, .03]. Overall, these analyses indicate that there was no trade-off between the OT and the PM task.

Table S1

*Test Statistics for the Bayesian Analyses Reported in Supplement 1*

| Study  and Condition | Dependent Variable | Predictors | Test statistic | 95% BCI | *BF*_01_ | *F* | *d.f.* | *p* |
| --- | --- | --- | --- | --- | --- | --- | --- | --- |
| 1 | Logarithmized RT | KSS before and after the PM task, PSQI | *R*^2^_corr_ = −.006 |  | 18.63 | 0.77 | 3, 114 | .513 |
|  | OT performance | KSS before and after the PM task, PSQI | *R*^2^_corr_ = +.030 |  | 02.79 | 2.30 | 3, 114 | .081 |
|  | Parameter *C*_1_ | PSQI | *b* = +.020 | [−.03, +.060] | 25.74 |  |  |  |
|  | Parameter *C*_1_ | KSS before the PM task | *b* = −.002 | [−.09, +.090] | 21.65 |  |  |  |
|  | Parameter *C*_1_ | KSS after the PM task | *b* = −.030 | [−.10, +.030] | 14.45 |  |  |  |
|  | Parameter *C*_2_ | PSQI | *b* = −.010 | [−.05, +.020] | 25.76 |  |  |  |
|  | Parameter *C*_2_ | KSS before the PM task | *b* = +.050 | [−.02, +.120] | 27.96 |  |  |  |
|  | Parameter *C*_2_ | KSS after the PM task | *b* = −.060 | [−.11, −.001] | 05.98 |  |  |  |
| 2 | Logarithmized RT | Posture (supine vs. upright), PSQI, interaction of posture and PSQI | *R*^2^_corr_ = +.007 |  | 09.26 | 1.23 | 3, 101 | .301 |
|  | OT performance | Posture (supine vs. upright), PSQI, interaction of posture and PSQI | *R*^2^_corr_ = −.010 |  | 20.09 | 0.61 | 3, 101 | .613 |
| Upright | Parameter *C*_1_ | PSQI | *b* = +.020 | [−.09, +.130] | 08.68 |  |  |  |
| Supine: | Parameter *C*_1_ | PSQI | *b* = −.040 | [−.16, +.090] | 07.19 |  |  |  |
| Upright: | Parameter *C*_2_ | PSQI | *b* = −.050 | [−.12, +.020] | 04.90 |  |  |  |
| Supine: | Parameter *C*_2_ | PSQI | *b* < +.010 | [−.06, +.060] | 17.25 |  |  |  |
| *Note.* BCI = Bayesian Confidence Interval. BF = Bayes Factor. RT = Reaction time; KSS = Karolinska Sleepiness Scale; PM = Prospective memory; PSQI = Pittsburgh Sleep Quality Index; OT performance = percent correct on the ongoing task; *C*_1_ = probability to detect a color match; *C*_2_ = probability to detect that colors do not match. | | | | | | | | |

Reference

Rummel, J., & Meiser, T. (2013). The role of metacognition in prospective memory: Anticipated task demands influence attention allocation strategies. *Consciousness and Cognition, 22,* 931–943. doi:[10.1016/j.concog.2013.06.006](https://doi.org/10.1016/j.concog.2013.06.006)

**Supplement 2: Response frequencies in Studies 1 and 2**

Table S2

*Response Frequencies Aggregated over Participants*

|  |  | Study 1 | | Study 2 | | | |
| --- | --- | --- | --- | --- | --- | --- | --- |
| Item type | Response |  | | Upright | | Supine | |
| Target, color match | “match” | 453 |  | 196 |  | 199 |  |
|  | “no match” | 79 |  | 45 |  | 73 |  |
|  | “PM” | 1,238 |  | 539 |  | 523 |  |
| Target, no color match | “match” | 56 |  | 29 |  | 24 |  |
|  | “no match” | 504 |  | 237 |  | 253 |  |
|  | “PM” | 1,210 |  | 514 |  | 518 |  |
| No target, color match | “match” | 14,695 |  | 6,072 |  | 5,950 |  |
|  | “no match” | 2,860 |  | 1,670 |  | 1,887 |  |
|  | “PM” | 145 |  | 58 |  | 113 |  |
| No target, no color match | “match” | 1357 |  | 488 |  | 605 |  |
|  | “no match” | 16,204 |  | 7,240 |  | 7,232 |  |
|  | “PM” | 139 |  | 72 |  | 113 |  |
| *Note.* “PM” = Prospective-memory response. | | | | | | | |

**Supplement 3: Frequentist analyses**

Table S3

*Results of Frequentist Statistical Tests Corresponding to Each of the Bayesian Tests Reported in the Article*

| Study | Frequentist test | Independent variable(s) | Dependent variable |  | Test statistic | *d.f.* | *p* | Effect size |
| --- | --- | --- | --- | --- | --- | --- | --- | --- |
| 1 | Multiple linear regression | KSS before and after the PM task, PSQI | PM hit rate |  | *F* = +2.40 | 3, 114 | +.071 | *R*^2^_corr_ = 0.04 |
| 2 | Two-tailed *t* test | Posture (supine vs. upright) | ESS |  | *t* = +1.05 | 102 | +.295 | *d* = 0.21 |
|  | Two-tailed *t* test | Posture (supine vs. upright) | PSQI |  | *t* = +1.71 | 102.35 | +.089 | *d* = 0.34 |
|  | One-sample, one-tailed *t* test (comparison value: 6) |  | PSQI | Upright:  Supine: | *t* = −6.40  *t* = −4.35 | 51  52 | < .001  < .001 | *d* = 0.89  *d* = 0.60 |
|  | 2 × 2 mixed ANOVA | Posture (supine vs. upright); Time of measurement (before vs. after the experiment) | KSS | Main effect of posture:  Main effect of time:  Interaction: | F = +1.26  F = 48.38  F = +5.56 | 1, 101  1, 101  1, 101 | +.264  < .001  +.020 | η_p_^2^ = 0.01  η_p_^2^ = 0.32  η_p_^2^ = 0.05 |
|  | MANOVA | Posture (supine vs. upright) | PVT: Lapses,  false alarms, logarithmized RTs |  | Λ = + .96  *F* = +1.36 | 3, 101 | +.259 | η_p_^2^ = 0.04 |
|  | Two-tailed *t* test | Posture (supine vs. upright) | Rate of correct responses in color-matching task |  | *t* = +1.03 | 103 | +.304 | *d* = 0.20 |
|  | Two-tailed *t* test | Posture (supine vs. upright) | PM hit rate |  | *t* = +0.54 | 103 | +.592 | *d* = 0.10 |
|  | Multiple linear regression | Posture (supine vs. upright), PSQI, interaction of posture and PSQI | PM hit rate | Regression weight of the interaction: | *F* = +1.14  *t* = −1.71 | 3, 104  100 | +.335  +.090 | *R*^2^_corr_ < 0.01  β = −.17 |
| *Note.* KSS = Karolinska Sleepiness Scale; PSQI = Pittsburgh Sleep Quality Index; PM = Prospective Memory; ESS = Epworth Sleepiness Scale; PVT = Psychomotor Vigilance Task. | | | | | | | | |

**Supplement 4: Correlation matrices**

Table S4.1

*Study 1*

|  | Retrospective Component of PM | PM Hits | OT Proportion Correct | KSS1 | KSS2 | PSQI |
| --- | --- | --- | --- | --- | --- | --- |
| Prospective Component of PM | *r* = −.07  [−.33, .19] | ***r* = .82**  **[.72, .89]** | ***r* = .31**  **[.12, .48]** | *r* = .15  [−.05, .34] | *r* = −.04  [−.24, .16] | *r* = −.02  [−.21, .18] |
| Retrospective Component of PM |  | ***r* = .24**  **[.01, .46]** | ***r* = .26**  **[.04, .46]** | *r* < .01  [−.21, .21] | *r* = −.17  [−.36, .05] | *r* = −.05  [−.25, .17] |
| PM Hits |  |  | ***r* = .36**  **[.19, .50]** | *r* = .15  [−.03, .32] | *r* = −.10  [−.27, .08] | *r* = −.03  [−.21, .15] |
| OT Proportion Correct |  |  |  | *r* = .03  [−.15, .20] | ***r* = −.20**  **[−.36, −.02]** | *r* = −.03  [−.20, .15] |
| KSS1 |  |  |  |  | ***r* = .44**  **[ .28, .57]** | ***r* = .27**  **[ .09, .42]** |
| KSS2 |  |  |  |  |  | ***r* = .26**  **[ .08, .42]** |

Table S4.2

*Study 2, Upright-Posture Group*

|  | Retrospective Component of PM | PM Hits | OT Proportion Correct | KSS1 | KSS2 | PSQI | ESS | PVT  false alarms | PVT logarithmized reaction times |
| --- | --- | --- | --- | --- | --- | --- | --- | --- | --- |
| Prospective Component of PM | *r* = −.25  [−.57, .11] | ***r* = .72**  **[ .50, .86]** | *r* = .09  [−.23, .38] | *r* = .02  [−.28, .32] | *r* = −.21  [−.49, .08] | *r* = .11  [−.19, .40] | *r* = .08  [−.22, .37] | *r* = .13  [−.17, .42] | *r* = −.19  [−.45, .11] |
| Retrospective Component of PM |  | *r* = .16  [−.18, .47] | ***r* = .34**  **[ .03, .59]** | *r* = .12  [−.20, .42] | *r* = .03  [−.27, .33] | *r* = .03  [−.27, .34] | *r* = −.06  [−.36, .25] | *r* = −.13  [−.41, .18] | *r* = .05  [−.26, .35] |
| PM Hits |  |  | *r* = .25  [−.03, .47] | *r* = .18  [−.10, .43] | *r* = −.24  [−.47, .04] | *r* = .13  [−.15, .38] | *r* = .08  [−.19, .34] | *r* = .07  [−.20, .33] | *r* = −.18  [−.42, .10] |
| OT Proportion Correct |  |  |  | *r* = .01  [−.26, .28] | *r* = −.23  [−.47, .04] | *r* = −.11  [−.36, .16] | *r* = −.27  [−.49, .01] | *r* = −.12  [−.37, .15] | *r* = −.08  [−.33, .19] |
| KSS1 |  |  |  |  | ***r* =** **.37**  **[ .10, .58]** | ***r* =** **.34**  **[ .07, .56]** | *r* = .06  [−.22, .32] | *r* = −.19  [−.44, .09] | *r* = .01  [−.26, .28] |
| KSS2 |  |  |  |  |  | ***r* =** **.28**  **[ .01, .51]** | *r* = .17  [−.10, .42] | *r* = −.16  [−.41, .11] | *r* = .18  [−.10, .42] |
| PSQI |  |  |  |  |  |  | *r* = .24  [−.04, .47] | *r* = .10  [−.18, .35] | *r* = .19  [−.09, .43] |
| ESS |  |  |  |  |  |  |  | ***r* =** **.31**  **[** **.04, .53]** | *r* = .10  [−.18, .35] |
| PVT false Alarms |  |  |  |  |  |  |  |  | ***r* =** **.51**  **[** **.27, .68]** |

Table S4.3

*Study 2, Supine-Posture Group*

|  | Retrospective Component of PM | PM Hits | OT Proportion Correct | KSS1 | KSS2 | PSQI | ESS | PVT  false alarms | PVT logarithmized reaction times |
| --- | --- | --- | --- | --- | --- | --- | --- | --- | --- |
| Prospective Component of PM | *r* = −.21  [−.54, .14] | ***r* = .62**  **[.33, .80]** | *r* = .13  [−.18, .41] | *r* = −.17  [−.44, .14] | *r* = −.20  [−.47, .11] | *r* = −.23  [−.50, .08] | *r* = .04  [−.27, .33] | *r* = −.08  [−.37, .27] | *r* = .02  [−.28, .32] |
| Retrospective Component of PM |  | ***r* = .37**  **[.08, .62]** | *r* = .20  [−.11, .47] | *r* = .17  [−.14, .44] | *r* = .17  [−.12, .45] | *r* = .04  [−.26, .33] | *r* = .23  [−.07, .49] | ***r* = −.47**  **[−.68, −.20]** | *r* = −.27  [−.53, .03] |
| PM Hits |  |  | ***r* = .28**  **[ .01, .50]** | *r* = −.06  [−.32, .21] | *r* = −.07  [−.32, .20] | *r* = −.20  [−.44, .07] | *r* = .15  [−.12, .40] | ***r* = −.46**  **[−.64, −.20]** | *r* = −.18  [−.42, .09] |
| OT Proportion Correct |  |  |  | *r* = −.16  [−.41, .11] | *r* = −.04  [−.30, .23] | *r* = −.06  [−.32, .21] | *r* = .04  [−.23, .30] | *r* = −.23  [−.46, .04] | ***r* =** −**.43**  **[−.61, −.17]** |
| KSS1 |  |  |  |  | ***r* = .61**  **[ .39, .74]** | *r* = .002  [−.26, .27] | *r* = .10  [−.18, .35] | *r* = −.12  [−.37, .16] | *r* = .19  [−.09, .43] |
| KSS2 |  |  |  |  |  | *r* = −.13  [−.38, .14] | *r* = .20  [−.07, .44] | *r* = −.19  [−.43, .09] | *r* = −.04  [−.30, .23] |
| PSQI |  |  |  |  |  |  | *r* = −.09  [−.35, .18] | *r* = .09  [−.19, .34] | *r* = −.02  [−.28, .25] |
| ESS |  |  |  |  |  |  |  | *r* = −.15  [−.40, .12] | *r* = .06  [−.21, .32] |
| PVT false Alarms |  |  |  |  |  |  |  |  | ***r* =** **.32**  **[ .05, .53]** |
| *Note.* Correlation matrices with 95% Bayesian Credibility Intervals (in brackets). Correlations with Bayesian Confidence Intervals that exclude zero (thus indicating evidence for a non-zero correlation) are highlighted in bold face. PM = Prospective Memory. OT = Ongoing Task. KSS1 = Karolinska Sleepiness Scale rating before performing the PM task. KSS2 = Karolinska Sleepiness Scale rating after performing the PM task. PSQI = Pittsburgh Sleep Quality Index. BF = Bayes Factor. ESS = Epworth Sleepiness Scale. PVT = Psychomotor Vigilance Task. | | | | | | | | | |
